# 4,5,4′-Trihydroxychalcone, 8,8′-(ethene-1,2-diyl)-dinaphtalene-1,4,5-triol and rutin from *Gynura segetum* inhibit phagocytosis, lymphocyte proliferation, cytokine release and nitric oxide production from phagocytic cells

**DOI:** 10.1186/s12906-017-1726-z

**Published:** 2017-04-11

**Authors:** Yuandani, Ibrahim Jantan, Khairana Husain

**Affiliations:** 1 Fakultas Farmasi, Universitas Sumatera Utara, 5 Jalan Almamater, USU-Kampus, Medan, 20155 Indonesia; 2 Drug and Herbal Research Center, Faculty of Pharmacy, Universiti Kebangsaan Malaysia, Jalan Raja Muda Abdul Aziz, 50300 Kuala Lumpur, Malaysia

**Keywords:** *Gynura Segetum*, 4*,*5,4'-Trihydroxychalcone, 8,8'-(Ethene-1,2-diyl)-dinaphtalene-1,4,5-triol, Rutin, HPLC analysis, Immunosuppressive effects

## Abstract

**Background:**

*Gynura segetum* is used traditionally to treat various ailments related to the immune system, which include cancer, inflammation, rheumatism, diabetes, hypertension, and viral infections but little studies have been carried out to validate their ethnopharmacological aspects. In this study the immunosuppressive effects of *G. segetum* and its constituents were investigated.

**Methods:**

Isolation of compounds from *G. segetum* leaves was conducted using vacuum liquid chromatography (VLC) and column chromatography (CC). Two new compounds, namely 4,5,4'-trihydroxychalcone and 8,8'-(ethene-1,2-diyl)-dinaphtalene-1,4,5-triol, together with stigmasterol and β-sitosterol were isolated from *G. segetum* methanol extract and their structures were determined spectroscopically. The presence of gallic acid and rutin in the extract was determined quantitatively by a validated HPLC method. *G. segetum* methanol extract and its constituents were investigated for their effects on chemotaxis, phagocytosis, β2 integrin (CD18) expression, and reactive oxygen species (ROS) of polymorphonuclear leukocytes (PMNs), lymphocytes proliferation, cytokine release and nitric oxide (NO) production of phagocytes.

**Results:**

All the samples significantly inhibited all the innate immune responses tested except CD 18 expression on surface of leukocytes. Among the samples, 8,8'-(ethene-1,2-diyl)-dinaphtalene-1,4,5-triol exhibited the strongest inhibitory on chemotaxis, phagocytosis, ROS and NO production. The compound exhibited exceptionally strong inhibitions on ROS and chemotaxis activities with IC_50_ values lower than the positive controls, aspirin and ibuprofen, respectively. 4,5,4'-Trihydroxychalcone revealed the strongest immunosuppressive activity on proliferation of lymphocytes (IC_50_ value of 1.52 μM) and on release of IL-1β (IC_50_ value of 6.69 μM). Meanwhile rutin was the most potent sample against release of TNF-α from monocytes (IC_50,_ 16.96 μM).

**Conclusion:**

The extract showed strong immunosuppressive effects on various components of the immune system and these activities were possibly contributed mainly by 4,5,4'-trihydroxychalcone, 8,8'-(ethene-1,2-diyl)-dinaphtalene-1,4,5-triol and rutin.

## Background

Modulation of the immune system is necessary in the treatment and management of those diseases due to defect or malfunction of the immune systems [1]. Immunosuppressive drugs inhibit the components of the immune system and they are used widely in the treatment of autoimmune diseases, inflammatory disorders and control pathological immune reaction of transplanted organs, while immunostimulant drugs activate the immune system and are widely used for the treatment of infectious diseases, cancer, allergy and immunodeficiency [2]. There are several chemical immunomodulators used in therapeutics but many of them have serious side effects. Aspirin and ibuprofen are among the non-steroidal anti-inflammatory drugs (NSAIDs) which can cause gastric and intestinal mucosal problems [3]. Corticosteroids, which have long been used as immunosuppressants have showed various side effects such as fluid retention, weight gain, diabetes, increased skin fragility and reduced bone marrow. Cyclosporin A, the most widely used immunosuppressant in transplanted rejection treatment, can cause nephrotoxicity, tremors, hypertension and gingival hyperthrophy. Interleukin-2 causes many side effects, such as hypotension, tachycardia and edema. New and safer drugs are required as alternatives and phytochemicals will continue to be a replenishable source of new and safe immunomodulatory agents [4].

In an effort to look for safer immunomodulators, many secondary metabolites such as terpenoids, phenolics, and alkaloids have been investigated for their ability to modulate the immune system. The extracts of many herbs such as *Panax ginseng, Tinospora cordifolia*, *Phyllanthus debilis*, *Centella asiatica Trigonella foenum graecum*, *Pouteria cambodiana*, *Picrorhiza scrophulariiflora* and *Garcinia mangostana* were able to upregulate or downregulate both innate and adaptive arms of the immune response [5–7]. The chemical constituents of these plants are potential new sources of immunomodulating agents. The assessment of immunological activities of phytochemicals can be based on their effects specifically on the various component and functions of the immune system. There have been a surge in interest to prospect for natural immunomodulators from plants which have been used traditionally to treat many immunological disorders.

The genus *Gynura* is an annual herb belonging to the family Asteraceae. It consists about 20 species, which is distributed in Africa, Australia, and various parts of Asia especially in Southeast Asia, more particularly in Indonesia, Malaysia, and Thailand [8]. Among them, *Gynura procumbens* known as ‘Sambung Nyawa’ and *G. segetum* known as ‘Daun Dewa’ are used traditionally to treat various ailments which include inflammation, rheumatism, cancer, viral infections, diabetes and hypertension [9, 10]. Many studies have been carried out to validate the pharmacological activities of *G. procumbens*. However, scientific studies on *G. segetum* were rarely reported. The leaf extract of *G. segetum* (Lour.) Merr. revealed potent anti-angiogenic activity which might lead to decrease tumor growth [11]. Phytochemical studies of the leaves of *G. segetum* showed the presence of flavonoids, tannins, saponins, terpenes, and alkaloids [9]. There is no effort yet to validate the traditional use of *G. segetum* leaves to treat diseases related to the immune system. Therefore, the present study was performed to determine the effects of the chemical constituents of the methanol extract of *G. segetum* leaves on phagocytic activities of polymorphonuclear leukocytes (PMNs), cytokine release, lymphocytes proliferation, and nitric oxide (NO) production of phagocytes.

## Methods

### Collection of plant material

*Gynura segetum* leaves was obtained from Yogyakarta, West Java, Indonesia in May 2012. A botanist of the Herbarium of Universiti Kebangsaan Malaysia (UKM) authenticated the plant material and a specimen (voucher number UKMB 29987) was deposited at the herbarium.

### Chemicals and reagents

Phorbol 12-myristatae 13-acetate (PMA), phytohemagglutinin (PHA), luminol (3-aminophthalhydrazide), Hanks Balance Salt Solution (HBSS++), RPMI 1640, sodium nitrite, Fluorescein isothiocyanate (FITC)-labelled opsonized *Escherichia coli,* lipopolysaccharides (LPS), ibuprofen (purity 99%), acetyl salicylic acid (purity 99%), prednisolone phosphate buffer saline tablet (PBS), dimethylsulfoxide (DMSO), gelatin from bovine skin, type B, dextran from *Leuconostoc mesenteroides*, ficoll, *N*-formyl-methionylleucylphenylalanine (fMLP), HEPES, and serum opsonized zymosan A from *Saccharomyces cerevisiae* were procured from Sigma (St Louis, MO, USA). Dexamethasone (purity 99%) and prednisolone (purity 99%) was purchased from Duopharma (Klang, Malaysia). Gallic acid and rutin (purity >98%) were obtained from ChromaDex (CA, USA). RAW 264.7 cells was purchased from ATCC. N-1(1-Naphthylethylenediamine dihydrochloride was obtained from Aldrich (USA). Trypan blue was obtained from FLUKA Analytica (UK). Penicillin/streptomycin (100×) and fetal calf serum (FCS) were procured from PAA Laboratories (USA). Isotype-matched Immunoglobulin-FITC (IgG1-FITC), CD18-FITC (anti-LFA-1β) and FACS lysing solution were purchased from Becton Dickinson, USA. Dulbecco’s modified Eagles Medium (DMEM) was obtained from Thermo Scientific (UK). Sulfanilamide was purchased from MP Biomedicals (France). Lympophrep was purchased from Fresenius Kabi Norge AS (Norway). Liquid scintillation cocktail (LSC) Solutions Ultima Gold and [^3^H]thymidine were obtained from Perkin Elmer (USA). A liquid scintillation counter (Perkin Elmer, USA), a CO_2_ incubator (Shell Lab, USA) and a microplate reader light microscope, (Thermo Scientific, UK) were also used in this assay. ELISA kits were obtained from Cayman (USA). Phagotest kit was purchased from Glycotope Technology, Germany. A Boyden 48-well chamber was procured from Neuro Probe (Cabin John, MD, USA). The flow cytometer BDFACS Canto II used was equipped with 488 nm argon ion laser. Xylene and haematoxylin used were obtained from BDH, UK. Chemiluminescence measurements were performed on a Luminoskan Ascent luminometer (Thermo Scientific, UK). Qualitative and quantitative analyses of samples were carried out using high pressure liquid chromatograph (Waters 2998) (Leitz Watzler, Germany). Determination of molecular weights was done by ESI-TOF-MS (Bruker MicroToF-Q 86, Switzerland). NMR 500 MHz (JOEL Ltd., Japan) was used to obtain ^1^H and ^13^C NMR spectra and internal standard used was TMS.

### Cell culture

RAW 264.7 murine macrophages cells were maintained in DMEM supplemented with 10% of FCS, and 100 units/mL each of of penicillin and streptomycin were grown at 37^0^ C and 5% CO_2_ in humidified air [12].

### Extraction and isolation of compounds

The leaf materials were allowed to dry under shade at room temperature. The dried material (700 g) was powdered, macerated and extracted with *n*-hexane, ethyl acetate, and methanol sequentially. The following extracts were obtained after removal of solvents under reduced pressure: *n*-hexane extract, 4.1 g, 0.57% *w*/w; ethyl acetate extract 12.4 g, 1.77% *w*/w; methanol extract 19.1, 2.73% *w*/w). The *n*-hexane extract (2.9 g) was applied to column chromatography with silica gel (40–63 μm) using *n*-hexane:EtOAc (9:1 to 1:9, *v*/v) and EtOAc:MeOH (9:0 to 0:9, *v*/v) as a gradient elution system to afford 30 mL each of 16 fractions. Fraction FH14 (675 mg) was purified by repeated silica gel column chromatography and recrystallization from *n*-hexane to afford compounds **1** (85.3 mg) and **2** (43.0 mg). The ethyl acetate extract (11 g) was subjected to vacuum liquid chromatography (VLC) on silica gel type 60HF_254_ (10–40 μm) with *n*-hexane:EtOAc (9:1 to 1:9, *v*/v) and EtOAc:MeOH (9:1 to 1:9, *v*/v) as a gradient system to yield 13 fractions. Fraction FE3 (250 mg) was further chromatographed on silica gel column with *n*-hexane:EtOA (1:1) as eluent to give white needle crystals, (**1**) (34.6 mg) and **2** (18.0 mg). Fraction FE8 (441 mg) was subjected to further chromatographed over sephadex LH-20 eluting with 100% CHCl_3_ and 100% MeOH to yield compound **3** (4.7 mg). Fraction FE9 (309 mg) was similarly treated as FE8 to yield compound **4** (5.4 mg). The methanol extract was chromatographed on sephadex LH-20 to yield a minute amount of **4** (1.8 mg). The purity of the isolated compounds were >98% as determined by NMR and MS-TOF data and based on their physicochemical properties.

### HPLC quantitative analysis of the methanol extract of *Gynura segetum*

HPLC quantitative analysis of the methanol extract of the plant was carried out using the modified method of Seow et al. [9]. The methanol extract and the reference standards (rutin and gallic acid) were dissolved in methanol to obtain 10 and 1 mg/mL solutions, respectively. The diluted solutions were filtered through Millipore Millex PTFE membrane (0.45 μm) (Maidstone, Kent, UK). HPLC qualitative and quantitative analyses of the filtered solutions were carried out by using a C-18 column Xbridge (250 mm × 4.6 mm i.d., 5 μm) (Waters, Ireland) with the following method: mobile phase: A. acetonitrile: B. water were eluted isocratically with 20% A and 80% B for 10 min at a flow rate of 1 mL/min. Detector used was PDA (Waters 2998), wavelength: 254 nm. Rutin and gallic acid were identified by comparing the UV-Vis spectra and retention times of the peaks and those of the standards. Quantification of the compounds in the extract was carried out by plotting calibration curves of each standard solution with four different concentrations (1000, 500, 250, and 125 μg/mL) versus the areas under the peaks. Precision, linearity, limits of quantification (LOQ) and detection (LOD) were performed to validate the reversed phase HPLC method. Linearity was determined by linear calibration analysis while the calibration curves were used to calculate the correlation coefficient (r^2^). Inter-assay and intra-assay validation was carried out to determine the precision of the method. LOQ and LOD were calculated from the RSD and slope (S) of the calibration curves using equations, LOQ = 3.3 x (RSD/S) and LOQ = 10 x (RSD/S).

### Isolation of polymorphonuclear cells (PMNs), peripheral blood mononuclear cells (PBMCs) and peripheral blood lymphocytes

Ten mL of human whole blood was collected from healthy persons (non-smokers who did not consume medicines or supplements and had fasted overnight) by aseptic vein puncture and placed in heparin-containing tubes. PMNs was isolated from the whole blood by Ficoll gradient separation whereas the isolation of peripheral blood monocytes (PBMCs) and peripheral blood lymphocytes were performed by using lymphoprep as described previously [13, 14]. Then, number of monocytes and lymphocytes were adjusted to 5 × 10^5^ cells/mL whereas PMNs was adjusted to 1 × 10^6^ cells/mL. Approval from the Human Ethical Committee of Universiti Kebangsaan Malaysia (no. FF/2012/Ibrahim/23-May/432-May 2012–August 2013) has been obtained for this study.

### Cell viability

Cytotoxity of the *G. segetum* extract and pure compounds on RAW 264.7, PBMC and peripheral blood lymphocytes cells were performed using MTT test [15], while viability test on PMNs was conducted using trypan blue [14].

### Chemotaxis

A Boyden chamber was used to perform PMNs migration assay with fMLP (10^−8^ M) as a chemoattractant [13]. The working concentrations of the extract and compounds were ranged from 3.125–50 or 6.25–100 μg/mL, respectively. Ibuprofen was used as a positive control, whereas chemoattractant buffer which contain equal amount of HBSS^++^ and DMSO was used as negative control. Xylene and haematoxylin fixed and stained the migrated cells and a light microscope was used to measure the cell migration distance.

### CD18 integrin expression of leukocytes

The assay was performed using flow cytometry which has been described by Mazzone et al. [16]. Test samples (compounds: 3.125 and 50 μg/mL; extract: 6.25 and 100 μg/mL) and 1 μL of LPS (0.25 μg/mL) were incubated in 100 μL of whole blood in a CO_2_ incubator (90 min; 37 °C). Then, all the sample tubes were placed in a box containing ice, simultaneously. Immunoglobulin IgG1-FITC (control) or CD18-FITC was mixed into the tubes. The red blood cells were lysed by incubating the tubes in the dark for 20 min after the addition of FACS lysing solution. Then, the mixture was centrifuged (250 g; 5 min at 4 °C) and washed with PBS (3×). The CD18 molecules expression was determined by flow cytometry and compared with the control. CD 18 expression was expressed as the percentage of CD18 complex [14, 16].

### Phagocytosis assay

The effect of *G. segetum* and its constituent on engulfment activity was perfomed as described in our previous paper [14]. The assay consist of five steps, which include labelling by incubating samples (extract: 6.25 and 100 μg/mL; compounds: 3.125 and 50 μg/mL) with whole blood and *E.coli.* After incubation, the quenching solution was added to the mixture to quench phagocytosis. Lysing and fixation were done by adding FACS-lysing solution, then centrifuged at 250 g, 2–5 °C for 5 min. Then, the leukocytes were washed with PBS and stained with DNA staining solution. The final step was determination of phagocytosis ability of PMNs and monocytes by flow cytometry.

### Reactive oxygen species (ROS) production

This assay was conducted according to our previous paper [14]. Samples (compounds: 3.125–50 μg/mL; extract: 6.25–100 μg/mL) were incubated with neutrophils. Opsonized zymosan or PMA was added to induce the cells and luminol (1 × 10^5^ M) was used as a probe. Then, the mixture was incubated (37 °C; 50 min) in a luminometer. The ROS inhibitory activity of samples was compared with acetylsalicylic acid as positive control and negative control (without sample). The results were expressed as chemiluminescence reading per luminometer unit (RLU) and then calculated as percentage of inhibition.

### Nitric oxide (NO) production

The NO production assay was conducted according to a previous paper by Yang et al. [12]. Briefly, RAW 264.7 macrophages cells (1 × 10^6^ cells/mL) were seeded in 96-well plates for 3 h. Then, cells were incubated wih test samples (compounds: 3.125–50 μg/mL; extract: 6.25–100 μg/mL) or dexamethasone (0.0004 to 4 μg/mL) for another 3 h, then stimulated with LPS (1 μg/mL). After incubation for 24 h at 37 °C, 5% CO_2,_ the production of nitric oxide was determined by measuring the quantity of nitrite in the medium using Griess reagent (0.1% naphthylethylenediamine dihydrochloride in 2.5% phosphoric acid and 1% sulfanilamide). One hundred μL of of Griess reagent was added to culture supernatant, then incubated for 10 min in a dark room. A microplate reader was used to measure absorbance at 550 nm and standard solution of sodium nitrite was used to calculate nitrite concentrations.

### Cytokine release assay

The enzyme linked immunosorbent assays (ELISA) were employed to determine the effects of the extract and compounds on the release of pro-inflammatory cytokines IL-1β and TNF-α in mononuclear cells, as described by Kumolosasi et al. [17]. The test samples (pure compounds: 3.125 to 50 μg/mL; extract: 6.25 to 100 μg/mL) or dexamethasone (0.0005 to 5 μg/mL) and LPS (1 μg/mL) were added to PBMCs suspension and incubated for 12 h at 37 °C, 5% CO_2._ The supernatant was transferred to 96-well plates after centrifugation of the mixtures at 300 x g for 10 min at 4 °C The appropriate ELISA-kit was used to assess the cytokine levels in supernatants of human blood cell cultures.

### Lymphocytes proliferation

The effect of the extract and pure compounds on lymphocytes proliferation was examined by measuring the incorporation of radioactive thymidine to newly synthesized DNA as written in previous paper [18]. Peripheral blood lymphocytes was incubated with samples (compounds: 3.125–50 μg/mL; extract: 6.25–100 μg/ml) or prednisolone (0.0005, 0.005, 0.05, 0.5, 5 μg/mL) and stimulated with PHA for 3 days in a CO_2_ incubator, followed by addition of [^3^H]Thymidine (0.5 μCi/well). After incubation for 18 h, harvesting was carried out using a cell harvester and a liquid scintillation counter was used to measure [^3^H]thymidine incorporation as count per minute (CPM).

### Statistical analysis

All the data were analysed using Statistical Package for Social Sciences (SPSS) version 15.0. The data were presented as mean ± standard error means (SEM). The IC_50_ values was calculated using Graph Pad Prism 6 software. A one-way analysis of variance (ANOVA) for multiple comparisons was employed for data analysis. *P* < 0.05 was considered to be different significantly.

## Results

### Isolation and structure identification of compounds

In this study, four compounds were isolated from the leaves of *G. segetum.* Identification of compounds was carrried out by spectroscopic technique. The physicochemical and spectroscopic properties were compared with literature values. Compounds **1** and **2** were obtained from the *n*-hexane and ethyl acetate extracts of *G. segetum,* whereas compounds **3** and **4** were isolated from ethyl acetate extract while compound **4** was also isolated at a low yield from the methanol extract.

**Fig. 1:**
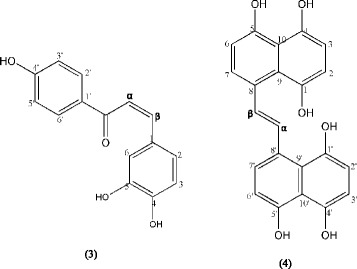
Structures of new compounds isolated from *Gynura segetum*

Compounds 1 and 2 were white crystalline needles with molecular ions at *m/z* 415.3561 and 413.3630 [M + H]^+^, respectively. Molecular formulas of compounds 1 and 2 were determined as C_29_H_50_O and C_29_H_48_O, respectively. The ^1^H- and ^13^C–NMR spectra confirmed that 1 and 2 were β-sitosterol and stigmasterol, respectively, as compared with the literature values [19]. Compound **3** was obtained as a red-orange solid. The HRESIMS spectrum showed a molecular ion peak [M + 2Na + H]^+^ at *m/z* 303.0853, corresponding to a molecular formula of a chalcone, C_15_H_12_O_4_. The ^1^H NMR spectrum displayed two proton peaks which were ortho coupled to each other based on their coupling constants at δ 6.72 (*d*, H-3′, H-5′, J = 8.5 Hz) and 7.78 (*d*, H-2′, H-6′, J = 8). It was supported by HSQC analysis which showed correlation between H-3′, H-5′ (δ 6.72) with C-3′, C-5′ (δ 113.9), respectively. HMBC spectrum also exhibited that the protons H-2′ showed ^2^J correlation with C-3′ (δ 113.9) and ^3^J coupling with C-4′ (δ 160.6). Meanwhile the protons H-3′ was ^3^J coupled with C-1′ (δ_C_ 124.0) and ^2^J correlation with C-4′ (δ 160.6). There were also three protons peaks which were attached to another ring at δ 7.35 (*d*, H-3, J = 8), 7.85 (*s*, H-6) and 8.04 (*d*, H-2, J = 7). The HSQC and HMBC spectrum used to show connectivities between protons and carbons indicated that the proton H-3 showed ^3^J coupling with C-5 (δ 125.9) and ^2^J correlation with C-2 (δ 120.4); the proton H-2 showed ^3^J coupling with C-β (δ 121.7) and ^3^J correlation with C-4 (δ_C_ 136.5); the proton H-6 exhibited ^2^J correlation with C-1 (δ 107.7) and C-5 (δ 125.9), ^3^J coupling with C-4 (δ 136.5). Signals at δ 7.09 (*d*, H-α, J = 7.5) and 7.10 (*d*, H-β, *J* = 7.5) demonstrated a cis correlation based on their coupling constant. The ^13^C NMR spectrum exhibited carbonyl carbons at δ 168.1 together with quartenary carbons at δ 107.7 (C-1), 124.0 (C-1′) and hydroxylated carbons at δ 136.5 (C-4), 125.9 (C-5), 160.6 (C-4′). Its UV spectrum showed strong absorption at 244.5 nm and weak absorption at 450 nm. Conclusively, **3** was identified as a new compound, 4,5,4′-trihydroxychalcone (Table 1). The structure of compound **3** is shown in Fig. 1.

**Table 1:** NMR spectroscopic data (500 MHz, MeOD) for compound **3** (δ in ppm)

Position	δC	δH, multiplicity (J in Hz)	HSQC	HMBC
1	107.7	-	-	-
2	120.4	8.04, d (7.0)	C-2, H-2	C-β, C-4
3	111.1	7.35, d (8.0)	C-3, H-3	C-2, C-5
4	136.5	-	-	-
5	125.9	-	-	-
6	131.4	7.85, s	C-6, H-6	C-1, C-5, C-4
1’	124	-	-	-
2’	131	7.78, d (8.0)	C-2’, H-2’	C-3’, C-4’
3’	113.9	6.72, d (8.5)	C-3’, H-3’	C-1’, C-4’
4’	160.6	-	-	-
5’	113.9	6.72, d (8.5)	C-5’, H-5’	C-1’, C-4’
6’	131.9	7.78, d (8.0)	C-6’, H-6’	C-5’, C-4’
C = O	168.1	-	-	-
α	120.5	7.09, d (7.5)	C-α, H-α	C-2, C-5
β	121.7	7.10, d (7.5)	C-β, H-β	C-3, C-4

Compound **4** was a white pale amorphous. The molecular ion peak [M + H_2_O + H]^+^, at *m/z* 395.0448, as shown in the HRESIMS spectrum, corresponded to a molecular formula of C_22_H_16_O_6._ The ^1^H–NMR (Table 2) showed signals at δ 6.78 (*d*, H-2, H-6, H-2′, H-6′, *J* = 7.5 Hz), 7.42 (*d*, H-7, H-7′, *J* = 8 Hz) and 7.86 (*d*, H-3, H-3′, *J* = 8.5 Hz) which were assigned as protons in aromatic ring. Signal at δ 6.78 was for four protons attached in four different rings, based on HSQC analysis. HMBC and NOESY correlations showed that these protons were connected to other proton peaks. Based on HMBC spectrum, the protons at δ 6.78 (H-6) showed ^2^ *J* coupling with C-5 (δ 122.6). Whereas, the H-3 and H-3′ proton at δ 7.86 displayed ^2^ *J* coupling with C-4 (δ 161.4) and C-4′ (δ 169.8), respectively. It was supported by NOESY correlations which showed interactions between H-2 and H-3, H-6 and H-7, H-2′ and H-3′ as well as H-6′ and H-7′. There was also one double bond which appeared at δ 6.29 (H-β) and 7.51 (H-α) which connected the dimer. The H-α proton, at δ 7.51 exhibited HMBC interactions with C-7′(δ 129.3) and C-1′ (δ 171.2). Meanwhile the proton at δ 6.29 (H-β) showed ^2^ *J* coupling with C-8 (δ 126.3) and NOESY correlations showed its interactions with H-7. The ^13^C NMR exhibited signals for hydroxylated carbons at δ 122.6, 130.8, 161.4, 159.2, 169.8, and 171.2. Meanwhile olefinic carbons were displayed at δ 116.6 and 143.5. The UV spectrum exhibited absorption peak at 251 nm. Thus, compound **4** was identified as a new compound 8,8′-(ethene-1,2-diyl)-dinaphtalene-1,4,5-triol. Figure 1 shows the chemical structure of compound **4**.

**Table 2:** NMR spectroscopic data (500 MHz, MeOD) for compound **4** (δ in ppm)

Position	δC	δH, multiplicity (*J* in Hz)	HSQC	HMBC	NOESY
1	159.2	-	-	-	-
2	115.3	6.78, *d* (7.5)	C-2	-	H-3
3	131.4	7.86, *d* (8.5)	C-3	C-4	**-**
4	161.4	-	-	-	-
5	122.9	-	-	-	-
6	115.3	6.78, *d* (7.5)	C-6	C-5, C-8	H-7
7	129.3	7.42, *d* (8)	C-7	C-1	H-6, H- β
8	126.3	-	-	-	-
9	114.3	-	-	-	-
10	114.3	-	-	-	-
1’	171.2	-	-	-	-
2’	115.3	6.78, *d* (7.5)	C-2’	-	H-3’
3’	131.4	7.86, *d* (8.5)	C-3’	C-4’	-
4’	169.8	-	-	-	-
5’	130.8	-	-	-	-
6’	115.3	6.78, *d* (7.5)	C-6’	-	H-7’
7’	129.3	7.42, *d* (8)	C-7’	C- α	H-6’
8’	128.3	-	-	-	-
9’	114.3	-	-	-	-
10’	114.4	-	-	-	-
β	116.6	6.29, *d* (16)	-	C-8	H-7
α	143.5	7.51, *d* (16)	-	C-7’, C-1’	-

#### 4,5,4′-Trihydroxychalcone (**3**)

Red-orange solid, Rƒ 0.5 in *n*-hexane:EtOAc (4:6), UV (MeOH), λ_max_ 244.5 and 450 nm; HRESIMS (positive-ion mode): *m/z* 303.0853 [M + 2Na + H]^+^ (Calcd. for C_15_H_12_O_4_ + 2Na + H 303.0575); see Table 1 for ^1^H NMR and ^13^C NMR spectroscopic datas.

#### 8,8′-(ethene-1,2-diyl)-dinaphtalene-1,4,5-triol (**4**)

White pale amorphous solid, Rƒ 0.5 in *n*-hexane:EtOAc (4:6), UV (MeOH), λ_max_ 251 nm; HRESIMS (positive-ion mode): *m/z* 395.0448 [M + H_2_O + H]^+^, (Calcd. for C_22_H_16_O_6_ + H_2_O + H, 395.1053); see Table 2 for ^1^H NMR and ^13^C NMR spectroscopic data.

### Quantitative analysis of the methanol extract of *G. segetum* by HPLC

**Fig. 2:**
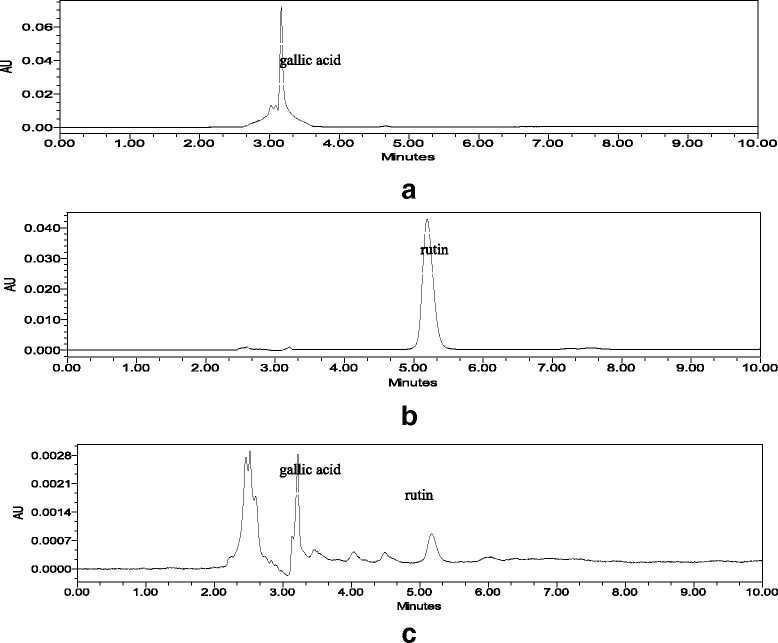
Representative HPLC chromatograms of (**a**) gallic acid, RT 3.078 min (**b**) rutin, RT 5.161 min (**c**) *Gynura segetum* methanol extract detected at the wavelength of 254 nm

The chromatogram of the methanol extract of *G. segetum* showed one major peak of gallic acid and minor peak of rutin, with retention times at 3.078 and 5.161 min, respectively. The peaks were compared to reference standards of gallic acid and rutin (Fig. 2). Quantification analysis showed the amounts of gallic acid and rutin as 191.4 and 24.1 μg/mL, respectively. Correlation coefficients (*r* ^2^) of 0.9995 and 0.9979 were obtained for gallic acid and rutin, respectively, as the calibration curves were linear over the concentration range of 1000–250 μg/mL. The % RSD values for interday assay precision of peak area and retention time were 3.4 and 0.5% for gallic acid and 6 and 8.6% for rutin, respectively. Whereas the % RSD values for intra-assay precision of peak area and retention time were 3.5 and 0.6% for gallic acid and 6 and 9.1% for rutin, respectively. These values demonstrated the reproducibility of the results. Limit of detection (LOD) and limit of quantification (LOQ) of rutin were found to be 0.44 and 1.34 μg/mL, respectively, while for gallic acid the values were 0.08 and 0.24 μg/mL, respectively.

### Chemotaxis assay

All the sample tested demonstrated strong inhibitory activity on PMNs migration without killing the cells at the highest doses of 50 and 100 μg/mL of *G. segetum* extract and pure compounds, respectively (≥ 90% cells were viable). Amongst the samples, 8,8′-(ethene-1,2-diyl)-dinaphtalene-1,4,5-triol (**4**) revealed the highest inhibitory activity (IC_50_ value of 2.22 μM) which was lower as compared to ibuprofen as a positive control (6.80 μM) (Table 3). The results indicate that 8,8′-(ethene-1,2-diyl)-dinaphtalene-1,4,5-triol (**3**) was more potent sample than ibuprofen.

**Table 3:** IC_50_ values (μg/mL) of chemotaxis, lymphocytes proliferation, NO and ROS inhibitory activities of *G. segetum* extract and its compounds on phagocytes (Mean ± SEM, *n* = 3)

Samples	Chemotaxis	Chemiluminescence				Nitric oxide	Lymph. Proliferation	Cytokines	
		Zymosan		PMA					
		Whole Blood	PMNs	Whole Blood	PMNs			TNF-α	IL-1β
*Gynura segetum*	2.03 ± 0.77	2.93 ± 1.62	2.63 ± 0.89	0.18 ± 0.12	1.41 ± 0.63	0.16 ± 0.03	3.25 ± 1.08	16.20 ± 3.94	2.72 ± 1.84
Rutin	2.53 ± 0.97	1.12 ± 2.32	0.08 ± 0.01	0.15 ± 0.34	0.05 ± 0.02	0.69 ± 1.98	2.26 ± 1.27	10.36 ± 6.98	6.95 ± 1.47
	(4.14 ± 1.09)	(1.95 ± 0.23)	(0.13 ± 0.01)	(0.25 ± 0.62)	(0.08 ± 0.02)	(1.13 ± 0.03)	(3.72 ± 2.08)	(16.96 ± 11.43)	(11.37 ± 2.41)
Gallic Acid	1.40 ± 0.02	1.70 ± 0.03	0.43 ± 1.02	0.87 ± 0.12	0.48 ± 0.01	4.13 ± 3.80	1.43 ± 0.65	13.92 ± 4.65	35.28 ± 3.62
	(8.22 ± 0.15)	(9.99 ± 0.12)	(2.53 ± 0.10)	(5.11 ± 0.15)	(2.82 ± 0.15)	(24.17 ± 12.50)	(8.43 ± 3.83)	(81.82 ± 1.87)	(207.38 ± 1.35)
4,5,4’-Trihydroxychalcone (**3**)	1.29 ± 0.89	4.92 ± 1.88	0.47 ± 0.03	5.14 ± 3.5	1.49 ± 0.38	0.39 ± 0.18	0.76 ± 0.36	18.87 ± 0.04	1.72 ± 0.77
	(5.04 ± 018)	(19.20 ± 3.55)	(1.84 ± 0.13)	(20.03 ± 6.04)	(5.86 ± 1.51)	(1.52 ± 0.73)	(2.96 ± 1.39)	(73.51 ± 0.14)	(6.69 ± 3.01)
Naphthelenetriol, (1,2-ethenedyl) (**4**)	0.84 ± 0.09	1.10 ± 1.12	0.02 ± 0.01	1.79 ± 0.80	0.05 ± 0.02	0.06 ± 0.03	4.59 ± 0.25	25.51 ± 11.12	16.76 ± 8.39
	(2.22 ± 0.33)	(2.93 ± 1.40)	(0.05 ± 0.02)	(4.77 ± 2.13)	(0.13 ± 0.08)	(0.15 ± 0.06)	(12.22 ± 0.67)	(67.84 ± 29.57)	(44.58 ± 22.33)
Ibuprofen	1.42 ± 0.14	–	–	–	–	–	–	–	–
	(6.80 ± 0.10)								
Aspirin	–	2.21 ± 0.79	1.91 ± 0.24	0.17 ± 0.01	0.14 ± 0.09	–	–	–	–
		(12.21 ± 1.10)	(10.52 ± 2.11)	(0.94 ± 0.05)	(0.77 ± 0.16)				
Prednisolone	–	–	–	–	–	–	0.038 ± 0.02	–	–
							(0.098 ± 0.066)		
Dexamethasone	–	–	–	–	–	0.0097 ± 0.002	–	0.77 ± 0.27	0.26 ± 1.02
						(0.025 ± 0.017)		(1.96 ± 0.44)	(0.67 ± 1.32)

IC_50_ values in μg/ml are in parentheses

### Inhibition of CD18 expression assay

Table 4 shows the effects of the compounds isolated from *G. segetum* and standard compounds (rutin and gallic acid) on CD18 complex expression on neutrophils and monocytes. The compounds displayed low inhibition of CD18 expression on the surface of phagocytes. The percentage of CD18 expression on the surface of phagocytes was similar to the untreated sample as positive control with the percentage of β2 integrin expression of 93.93 and 84.79% for neutrophils and monocytes for positive control at 37^0^ C, respectively.

**Table 4:** Percentage of CD18 expression activity (%) of neutrophils and monocytes at various concentrations of *G. segetum* extract and its compounds (Mean ± SEM, *n* = 3)

Sample (μg/mL)	Neutrophils				Monocytes			
	100	50	6.25	3.125	100	50	6.25	3.125
*G. segetum* extract		82.1 ± 11.9		90.0 ± 5.2		78.0 ± 8.5		84.8 ± 7.4
Rutin		88.75 ± 0.49		99.35 ± 0.64		91.60 ± 1.84		91.20 ± 1.56
Gallic Acid	81.33 ± 1.80		88.77 ± .41		68.60 ± 1.20		76.50 ± 2.18	
4,5,4’-trihydroxy chalcone **(3)**		94.60 ± 0.42		93.80 ± .42		96.25 ± 2.33		97.60 ± 0.85
8,8’-(ethene-1,2-diyl)-dinaphtalene-1,4,5-triol (**4**)		97.80 ± 2.30		93.10 ± 4.20		79.90 ± 4.50		92.30 ± 5.60

### Phagocytosis activity

**Fig. 3:**
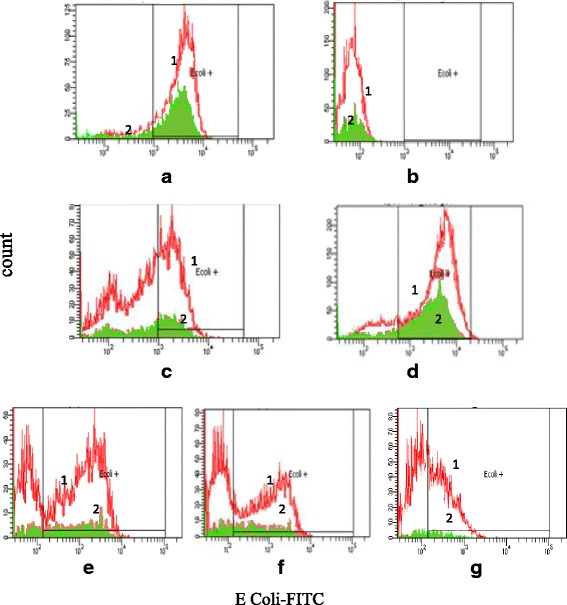
Representation of *E.coli* engulfment by neutrophils (1) and monocytes (2). **a** positive control **b** negative control, **c** *Gynura segetum* extract **d** Rutin **e** Gallic acid **f** 4,5,4’-Trihydroxychalcone (**3**) **g** 8,8’-(ethene-1,2-diyl)-dinaphtalene-1,4,5-triol (**4**)

The number of neutrophils and monocytes that engulf pathogen were assayed by flow cytometry. Gallic acid showed the lowest inhibitory activity on immunoglobulin and complement opsonized *E. coli* engulfment by phagocytes. Amongs the samples, 8,8′-(ethene-1,2-diyl)-dinaphtalene-1,4,5-triol (**4**) was the most potent sample in inhibiting phagocytosis ability with percentage of the phagocytizing cells of 44.90 and 47.67% for monocytes and PMNs, respectively. The tubes without sample incubated at 37^o^ C was used as a positive control while tubes at 0^o^ C was used negative control (Fig. 3).

### Inhibition of reactive oxygen species (ROS) generation

Effects of all the samples on both pathway were investigated using opsonized zymosan and PMA as stimuli. Preliminary evaluation of the samples on the whole blood displayed that all the compounds exhibited strong inhibitory activity upon activation by PMA and zymosan. The samples were further examined for their effects on the respiratory burst of neutrophils. Rutin and 8,8′-(ethene-1,2-diyl)-dinaphtalene-1,4,5-triol (**4**) revealed similar ROS inhibitory against PMN upon activation by PMA and zymosan with IC_50_ values of 0.08 and 0.13 μM for rutin, respectively and 0.13 and 0.05 μM for 8,8′-(ethene-1,2-diyl)-dinaphtalene-1,4,5-triol (**4**), respectively (Table 3).

### Inhibition of nitric oxide (NO) production

Table 3 shows the inhibitory activity of standard compounds and isolates from *G. segetum* on nitric oxide production from RAW 264.7 macrophages cells. All the samples tested revealed significant inhibition with percentage inhibition at highest concentration ranging from 73.86 to 81.57%. As shown in Table 4, 8,8′-(ethene-1,2-diyl)-dinaphtalene-1,4,5-triol (**4**) depicted the strongest NO inhibitory activity with an IC_50_ value of 0.15 μM. However, its IC_50_ value was higher than that of dexamethasone as positive control (0.025 μM).

### Cytokine release

As shown in Table 3, all the compounds tested depicted significant inhibition against proinflammatory cytokines release from peripheral blood mononuclear cells at the highest concentration of 100 μg/mL for gallic acid and 50 μg/mL for others samples. Amongst the compounds, 4,5,4′-trihydroxychalcone (**3**) demonstrated the highest inhibition on IL-1β release (IC_50_ value of 6.69 μM). Meanwhile rutin was the most potent sample against TNF-α release from monocytess with an IC_50_ value 16.96 μM. Both compounds might be contributing to the inhibitory activity of *G. segetum* extract on cytokine release.

### Lymphocytes proliferation

Table 3 shows the lymphocytes anti-proliferation activity of standard compounds present in *G. segetum* as well as its isolates. Among the samples, 4,5,4′-trihydroxychalcone (**3**) was the most potent samples against lymphocytes proliferation (IC_50_ value of 1.52 μM) which was comparable to prednisolone (IC_50_ value of 0.098 μM) as positive control. Meanwhile gallic acid and rutin, significantly inhibited lymphocytes proliferation after stimulation by a mitogen, PHA (IC_50_ values of 0.96 and 2.19 μg/mL, respectively).

## Discussion

Phytochemical studies on *G. segetum* leaves have resulted in the isolation of four compounds. Amongst them, 4,5,4′-trihydroxychalcone (**3**) is a new naturally occuring compound and this is the first report of its isolation from natural resource, while 8,8′-(ethene-1,2-diyl)-dinaphtalene-1,4,5-triol (**4**) was a new compound. A validated HPLC method was used to quantify the amounts of rutin and gallic acid in *G. segetum* methanol extract. The cytotoxicity of the plant extract and its constituents was performed using trypan blue and MTT tests. At the highest doses of 50 and 100 μg/mL of *G. segetum* extract and pure compounds, respectively, the cells showed ≥90% viability.

The ability of *G . segetum* extracts and its constituents to modulate the immune response was determined by various immunological assays which represent different lineages of immune response. The plant samples were investigated for their effects on chemotaxis, phagocytosis, CD18 expression, and ROS of PMNs, lymphocytes proliferation, cytokine release and NO production of phagocytes. Phagocytic activity consists of several steps, that is, chemotaxis, adherence through the interaction of β_2_ integrins with vascular endothelial cells, and engulfment of the pathogen, followed by intracellular destruction. Microbicidal mechanism involves generation of toxic molecules, such as hypochlorous acid (HOCl^−^), hydroxyl radicals (OH^−^), singlet oxygen and peroxynitrite [20]. Lymphocytes are cells that are most responsible to initiate and perform adaptive immunity. Cytokines also play important role in both innate and acquired immune responses, for example IL-1 and TNF which increase leukocyte/endothelial cell adhesion by increased expression of ligands for integrins [21].

*G. segetum* and its constituents demonstrated strong inhibition on PMN chemotaxis. The results indicate that 8,8′-(ethene-1,2-diyl)-dinaphtalene-1,4,5-triol (**4**) was more potent than ibuprofen. Previous study reported the ability of ibuprofen to reduce the interaction between fMLP with its receptor, hence decreasing the migration of PMNs to the site of interaction [22]. The results suggest that *G. segetum* and its compounds could also decrease the interaction of fMLP with the receptor. The extract and compounds displayed low inhibition of CD18 expression on the surface of phagocytes. According to Luster [23], NO by itself downregulates the expression of integrin on neutrophils, such as CD11a/CD18 (LFA-1). In this study, all the samples showed strong inhibition on NO production. The less amount of NO might be the reason for low inhibition of the expression of integrin on human phagocytes.

The engulfment activity is initiated by recognition of microbes by phagocytes. Complement surface receptors on neutrophils easily recognize complement-opsonized microbes. Meanwhile, immunoglobulin-coated microbes are detected by PMNs receptors specific for the Fc-region of immunoglobulin. Interaction of the immunoglobulin and complement with their receptors at the PMN surface initiates phagocytosis of pathogens [24]. The results suggest most of the samples, especially 8,8′-(ethene-1,2-diyl)-dinaphtalene-1,4,5-triol (**4**) has the ability to block those receptors, hence phagocytosis was inhibited.

ROS is produced through independent and dependent receptor pathway. The ROS inhibitory activities of rutin and 8,8′-(ethene-1,2-diyl)-dinaphtalene-1,4,5-triol (**4**) were higher than aspirin as positive control upon activation by zymosan and PMA (IC_50_ values of 0.77 and 10.52 μM, respectively). It was reported that aspirin inhibited luminol-amplified chemiluminescence of human neutrophils [25]. Opsonized zymosan stimulated the activity of the enzyme NADPH oxidase by receptor-mediated, whereas PMA activated the enzyme through receptor-independent process [26]. The results suggest all the samples were able to inhibit the ROS production through both pathways. The free phenolic hydroxyl group present in the compounds enhanced the ability to scavenge free-radicals [27].

NO production of macrophages by the enzymatic of inducible nitric oxide synthase (iNOS) has been reduced after exposure to *G. segetum* extract and its compounds. The results indicate that all the samples inhibited the enzymatic activity of iNOS. The results suggest 8,8′-(ethene-1,2-diyl)-dinaphtalene-1,4,5-triol (**4**) has major contribution on the NO inhibitory activity of *G. segetum* extract. Except gallic acid, other compounds tested also exhibited similar activity but quite lower than those of 8,8′-(ethene-1,2-diyl)-dinaphtalene-1,4,5-triol (**4**) and dexamethasone.

Levels of cytokine were increased upon activation of MAPK cascade by LPS which regulates both activator protein-1 (AP-1)-associated gene transcription and NF-ĸB [28]. Another study also reported that corticosteroids act as native inhibitors of IL-l synthesis by mononuclear phagocytes by suppressing biosynthesis of IL-1α and IL-1β at the transcriptional level [29]. Hence, the inhibitory activity of *G. segetum* extract and its compounds might be due to blockage of biosynthesis pathway at the transcriptional level. IL-1 enhanced CD4+ T-cell proliferation, promoted B-cell growth and differentiation at low locally produced levels [30]. Inhibition on IL-1 release by *G. segetum* and its compounds consequently inhibited lymphocytes proliferation. Prednisolone was used as a positive control which inhibited lymphocytes proliferation as described previously [31].

## Conclusion

The HPLC analysis of the methanol extract of *G. segetum* led to identification of rutin and gallic acid. Chromatographic technique led to the separation of two new compounds, 4,5,4′-trihydroxychalcone (**3**) and 8,8′-(ethene-1,2-diyl)-dinaphtalene-1,4,5-triol (**4**) The in vitro studies on phagocytes showed the methanol extract of *G. segetum* and its constituents exhibited significant inhibitory activity of migration of neutrophils towards chemoattractant. All the samples could inhibit ROS production of PMA and zymosan-stimulated PMNs with 8,8′-(ethene-1,2-diyl)-dinaphtalene-1,4,5-triol (**4**) as the most potent sample in inhibiting ROS production as well as chemotaxis activity of PMNs. 8,8′-(ethene-1,2-diyl)-dinaphtalene-1,4,5-triol (**4**) also demonstrated the strongest phagocytosis and NO inhibitory activities. All the samples exhibited weak inhibition on CD18 expression on surface of leukocytes. Amongst the samples, 4,5,4′-trihydroxychalcone (**3**) demonstrated the highest inhibitory activity on IL-1β release, while rutin was the most potent sample against TNF-α release from monocytes. Conclusively, the methanol extract of *G. segetum* and its constituents could suppress significantly every lineages except CD18 expression with 8,8′-(ethene-1,2-diyl)-dinaphtalene-1,4,5-triol (**4**) being the most potent sample in most activities. The high inhibitory activity of *G. segetum* extract could be due to the presence of 8,8′-(ethene-1,2-diyl)-dinaphtalene-1,4,5-triol (**4**) without excluding contribution of 4,5,4′-trihydroxychalcone, rutin and gallic acid as well as other constituents. The naphtalene derivative could be a lead compound for further development into an immunotherapeutic agent which can be a potential alternative to cyclosporin A. However, further studies have to be performed to investigate their molecular effects.

## Abbreviations

DMEM: Dulbecco’s modified Eagles Medium
FCS: Fetal calf serum
FITC: Fluorescein isothiocyanate
fMLP: N-formyl-methionylleucylphenylalanine
HBSS: Hanks Balance Salt Solution
IgG1: Isotype-matched Immunoglobulin
IL: Interleukin
LPS: Lipopolysaccharides
LSC: Liquid scintillation counter
NO: Nitric oxide
NSAIDs: Non-steroidal anti-inflammatory drugs
PBMCs: Peripheral blood mononuclear cells
PHA: Phytohemagglutinin
PMA: Phorbol 12-myristatae 13-acetate
PMNs: Polymorphonuclear leukocytes
ROS: Reactive oxygen species
TNF: Tumor necrosis factor
